# Melatonin attenuates inflammation, remodeling, and oxidative stress in experimental models of asthma and COPD

**DOI:** 10.3389/fimmu.2026.1915509

**Published:** 2026-09-21

**Authors:** Cristiane de Cássia Ribeiro, Marcela de Sousa João, Francine Maria de Almeida, Silvia Fukuzaki, Suellen Karoline Moreira Bezerra, Vitório Crema Scheffer, Fernanda Roberto, Fabíola Santos Zambon Robertoni, Alex Ferreira da Silva, Walcy Paganelli Rosolia Teodoro, Renato Fraga Righetti, Leandro do Nascimento Camargo, Iolanda de Fátima Lopes Calvo Tibério, Edna Aparecida Leick

**Affiliations:** 1 Department of Clinical Medicine, Faculty of Medicine, University of Sao Paulo, Sao Paulo, SP, Brazil; 2 Rehabilitation Service, Hospital Sírio-Libanês, Sao Paulo, Brazil

**Keywords:** asthma, COPD, experimental model, inflammation, melatonin, oxidative stress, pulmonary function

## Abstract

**Introduction:**

Asthma and chronic obstructive pulmonary disease (COPD) are characterized by persistent airway inflammation, oxidative stress, and structural remodeling, leading to progressive impairment of lung function. Melatonin has anti-inflammatory and antioxidant properties and may represent a promising therapeutic strategy for chronic airway diseases.

**Objective:**

To evaluate the therapeutic effects of melatonin on airway hyperresponsiveness, inflammation, oxidative stress, and remodeling in experimental models of asthma and COPD.

**Methods:**

Forty male BALB/c mice were allocated into five groups (n = 8/group): SAL, OVA, OVA+MEL, ELA, and ELA+MEL. Asthma and emphysema were induced by ovalbumin and elastase, respectively. Melatonin (15 mg/kg, intraperitoneally) was administered from days 22 to 27. Respiratory mechanics, bronchoalveolar lavage fluid, immunohistochemistry, and gene expression analyses were performed to evaluate inflammatory cytokines, remodeling markers, and oxidative stress-related proteins.

**Results:**

Melatonin significantly attenuated airway hyperresponsiveness in the asthma model, reducing respiratory mechanical parameters, inflammatory cell infiltration, and eosinophilia. In both asthma and emphysema models, melatonin markedly decreased the expression of pro-inflammatory cytokines, remodeling markers (MMP-9, MMP-12, TIMP-1, and TGF-β), and oxidative stress-related mediators (NF-κB and iNOS). These findings were further supported by gene expression analyses, demonstrating broad suppression of inflammatory cytokines following treatment.

**Conclusion:**

Melatonin attenuated inflammation, oxidative stress, and airway remodeling while improving lung function, particularly in experimental asthma. These findings highlight melatonin as a promising adjunctive therapeutic strategy for chronic inflammatory lung diseases by simultaneously targeting multiple pathogenic pathways.

## Introduction

Asthma is a heterogeneous respiratory condition generally characterized by chronic airway inflammation. Clinically, it is defined by the presence of respiratory symptoms such as wheezing, dyspnea, chest tightness, and cough, which vary in time and intensity and are associated with variable expiratory airflow limitation. This definition was established by consensus, considering the typical clinical and pathophysiological features of asthma observed before the initiation of treatment, which distinguish it from other respiratory diseases. However, as the disease progresses, airflow limitation may become persistent. Asthma is a heterogeneous disease, usually characterized by chronic airway inflammation (1).

Chronic Obstructive Pulmonary Disease (COPD) represents a major global public health problem and is the fourth leading cause of death worldwide. In 2021, it was responsible for approximately 3.5 million deaths, accounting for about 5% of all global mortality. In 2019, an estimated 392 million cases of COPD were reported globally (2).

Chronic airway inflammation is a highly relevant feature in the pathophysiology of COPD (3, 4). This persistent inflammation causes structural changes and narrowing of the bronchial lumen. Destruction of the pulmonary parenchyma leads to loss of alveolar attachments in the small airways and a reduction in the elastic recoil of the lungs. These alterations impair the ability of the airways to remain open during expiration (4). Pulmonary diseases are now recognized as a major health concern and rank among the leading causes of death worldwide. They are associated with high hospitalization costs, multiple comorbidities, and a significant impact on individuals’ functional capacity. In this context, several authors have investigated the use of melatonin, which exerts multiple physiological effects as an antioxidant, anti-inflammatory, immunoneuromodulatory, and anti-ferroptotic agent (5, 6).

Circulating melatonin can reach all body tissues and is capable of crossing the blood-brain barrier to modulate brain activity (7).

In recent years, melatonin has been studied for its physiological effects (such as regulation of the sleep–wake cycle, sleep promotion, and seasonal adaptation) as well as its therapeutic potential in cardiovascular and bone metabolism, renal function, the gastrointestinal system, and anticancer effects.

In the respiratory tract, melatonin may influence airway smooth muscle tone and exert immunomodulatory functions that could be potentially beneficial for asthma treatment. In some *in vitro* studies using human bronchial smooth muscle, melatonin induced concentration-dependent relaxation (8).

Studies have shown that intraperitoneal administration of melatonin at 10 mg/kg significantly inhibited NF-κB expression and downregulated inducible nitric oxide synthase (iNOS) activity in lung tissues, as well as markedly reduced nitric oxide production in bronchoalveolar lavage fluid in an experimental model of bronchial asthma. These authors concluded that melatonin may reduce airway hyperresponsiveness and inflammation in an ovalbumin-induced asthma model (9). In a subsequent study, the same researchers found that melatonin significantly inhibited connective tissue growth factor expression and reduced mucus area in the ovalbumin-induced asthma mouse model, showing activity comparable to that of dexamethasone (10).

Furthermore, a double-blind, randomized, placebo-controlled study conducted in adult women with asthma demonstrated that oral administration of 3 mg of melatonin for 28 days improved subjective sleep quality in patients with mild to moderate asthma compared to the placebo group (8).

In-sik Shin et al. investigated the effects of melatonin on pro-inflammatory cytokines and on the activity and expression of matrix metalloproteinase-9 (MMP-9) in BEAS-2B cells stimulated by tumor necrosis factor (TNF-α). Melatonin administration significantly reduced inflammatory cell counts, airway hyperresponsiveness, and immunoglobulin (Ig) E levels, along with reductions in interleukins IL-4, IL-5, and IL-13. Melatonin attenuated airway inflammation and mucus production in lung tissue and significantly suppressed elevated MMP-9 expression and activity in OVA models. In BEAS-2B cells stimulated by TNF-α, melatonin treatment significantly reduced pro-inflammatory cytokine levels and decreased MMP-9 expression and activity. These findings indicate that melatonin effectively suppressed allergic asthma induced by OVA challenge (11).

Melatonin, an indoleamine, has the ability to activate antioxidant enzyme mechanisms across different tissues, regulate smooth muscle tone, and influence immune responses, being highly efficient in scavenging free radicals due to its strong antioxidant properties (12, 13). In 2023, Qin et al. reported that melatonin restored body weight, reduced the lung weight-to-body weight ratio, alleviated histopathological lung injury, and decreased cytokine levels (tumor necrosis factor-α [TNF-α], interleukin [IL]-12p70, IL-17, and IL-10) in bronchoalveolar lavage fluid in a murine model of lipopolysaccharide (LPS)-induced acute lung injury. Moreover, melatonin reduced the expression of the activation marker CD69 and decreased the secretion of pro-inflammatory cytokines (TNF-α, IL-12p70, and IL-17) following LPS challenge (14).

Viana et al., after completing a randomized controlled clinical trial, concluded that melatonin supplementation combined with rehabilitation over a 12-week period may improve functional capacity, health status, and quality of life in patients with COPD (15).

Given the limited evidence regarding melatonin’s effects in asthma and COPD, this study aimed to investigate the therapeutic effects of melatonin on bronchial hyperresponsiveness, airway inflammation, and remodeling in experimental models of asthma and COPD.

## Materials and methods

This project was approved by the Research Ethics Committee of the Hospital das Clínicas of the Medical School of the University of São Paulo (protocol number 1430/2020). This work was developed in the Laboratory of Experimental Therapy I (LIM 20) of the Faculty of Medicine of the University of São Paulo.

### Experimental groups

Forty male BALB/C mice from the Faculty of Medicine of the University of São Paulo were utilized in accordance with the Guideline to Care and Use of Laboratory Animals, published by the National Institutes of Health (NIH publication 85-23, revised in 1985). Forty male BALB/c mice were divided into five experimental groups (n = 8 per group): SAL, OVA, OVA+MEL, ELA, and ELA+MEL,

1. control saline SAL GROUP: mice received inhalations with a sterile saline solution (n=8);
2. OVA GROUP: mice received IP and inhalations of an ovalbumin solution (n=8);
3. OVA + MEL GROUP: mice received IP and inhalations of an ovalbumin solution and treatment with melatonin solution (n=8);
4. ELA GROUP: mice received IP porcine pancreatic elastase solution (n=8);
5. ELA + MEL GROUP: mice received IP porcine pancreatic elastase and treatment with melatonin solution (n=8).

### Ovalbumin-induced asthma mice model

The sensitization protocol lasted for 28 days. On days 1 and 14, the BALB/c mice received a solution of 50 µg of OVA (Sigma-Aldrich) and 6 mg of Al (OH)_3_ adjuvant (Pepsamar, Sanofi) intraperitoneally (i.p.) in a total volume of 0.2 mL (Synthelabo SA, Rio de Janeiro, Brazil). On days 21, 23, 25, and 27, the animals were submitted to inhalation for 30 minutes, in an ultrasonic nebulizer (US-1000, ICEL, São Paulo, Brazil) coupled to an acrylic box (30 × 15 × 20 cm), diluted in 0.9% NaCl at 1% concentration. At the same time, the control group was administered a saline solution (NaCl 0.9%) i.p. and was exposed to 0.9% saline aerosol for 30 minutes for the inhalation challenge. On the 28^th^ day, the mechanics of the respiratory system and bronchoalveolar lavage were performed (16).

### Elastase-induced emphysema mice model

For porcine pancreatic elastase (PPE) (E1250–500 mg Elastase from porcine pancreas Type I, ≥4 units/mg protein, 41.7 mL, 12 mg protein/mL; 5 units/mg Protein; Sigma Aldrich, St. Louis, MO, USA) instillation, the mice were anesthetized with inhaled isoflurane (Isofurine^®^ 1 mL/mL, Cristália LTDA, Itapira, SP, Brazil). The experimental animals received intratracheal instillation of 25 U PPE/100 g body weight dissolved in 40 µL of normal saline, on day 21 of the protocol, as previously described by Ikeda et al., 2014 (17). All animals were evaluated after seven days of elastase treatment.

### Melatonin treatment groups

The mice received intraperitoneal injections of 15 mg/kg of melatonin on days 22, 23, 24, 25, 26 and 27 (Melatonin – Sigma - Aldrich), 1 h before the OVA challenge which was described previously by Shin et al., 2014 (11).

### Determination of lung hyperresponsiveness to methacholine

Twenty-four hours after the induction of the experimental disease models, animals were anesthetized with thiopental (50 mg/kg, i.p.) and underwent tracheostomy. A plastic cannula was inserted into the trachea through the incision and secured in place. The animals were then connected to a mechanical ventilator designed for small animals (FlexiVent, Scireq, Montreal, Canada). Ventilation parameters included a tidal volume of 10 mL/kg, a respiratory rate of 120 breaths per minute, and a sinusoidal inspiratory flow pattern. To suppress spontaneous respiratory efforts, pancuronium (0.2 mg/kg, i.p.) was administered.

The generated pressure values were recorded, and airway impedance (pressure/flow) was calculated as a function of the different oscillatory frequencies applied during the measurement. Using a 75% over lapping window on the 16-second signal, three 8-second blocks were used to calculate the oscillatory mechanics parameters, using the equation: Z (f) = Raw + i (2pf) Law + [Gtis = iHtis]/(2pf). These values were used to calculate the respiratory system basal and maximum resistance (Rrs) and elastance (Ers) after methacholine aerosol (3, 30, and 300mg/ml for 1min) administration. The resistance and elastance of the respiratory system were calculated using the equation of respiratory system movement. We described the lung model using the following parameters: resistance of larger airways (Raw), resistance of smaller airways or tissues (Gtis), and elastance of lung tissues (Htis). After respiratory mechanics evaluation, animals were euthanized by abdominal aorta exsanguination (18).

### Analysis of bronchoalveolar lavage fluid

Bronchoalveolar lavage was performed by infusing 0.5 mL of saline solution three consecutive times (total volume of 1.5 mL) through the tracheal cannula using a syringe. The recovered fluid was centrifuged at 1,000 rpm, 5 °C, for 10 minutes, with an average recovery rate of 80%. The cell pellet was resuspended in 200 µL of saline. Total cell counts were performed using light microscopy and a Neubauer hemocytometer (400x magnification). For differential cell counts, 100 µL of the BAL fluid were cytocentrifuged at 450 rpm for 6 minutes. After air drying, the slides were stained using the Diff-Quick Reagent (Biochemical Sciences Inc., Swedesboro, NJ). Differential leukocyte counts were determined by evaluating 300 cells per slide, and classification of neutrophils, eosinophils, lymphocytes, and macrophages was performed based on standard hematological criteria, using oil immersion objective microscopy (1000x) (19).

### Histology and immunohistochemistry

After fixation, the material was subjected to the usual histological techniques with paraffin, to obtain sections of 4 µm thickness, and the slides were stained as described below. Diluted primary antibodies (**Table 1**) were applied to the tissue sections, and the slides were incubated overnight (18–20 hours) in a humidified chamber. On the following day, slides were rinsed with PBS and incubated with the appropriate secondary antibody from the Vectastain ABC Kit system (Vector Elite: PK-6105 anti-goat, PK-6101 anti-rabbit, or PK-6102 anti-mouse). Immunoreactive cells were visualized using 3,3′-diaminobenzidine (DAB) chromogen (Sigma Chemical Co., St. Louis, MO, USA). The slides were then counterstained with Harris hematoxylin (Merck, Darmstadt, Germany), mounted with Entellan microscopy resin (Merck, Darmstadt, Germany), and subjected to morphometric analysis as described below (17, 20).

**Table 1:** Description of antibodies used for immunohistochemistry analysis.

Marker	Dilution	Primary antibody	Specification
IL-2	1:400	Anti-mouse	SC-133118 Mouse Monoclonal; Sta Cruz Biotechnology, CA, USA
IL-4	1:200	Anti-mouse	SC-53084 Mouse Monoclonal; Sta Cruz Biotechnology, CA, USA
IL-5	1:300	Anti-mouse	SC-398334 Rabbit Polyclonal; Sta Cruz Biotechnology, CA, USA
IL-6	1:400	Anti-mouse	LS-C 57315 Mouse Monoclonal; Sta Cruz Biotechnology, CA, USA
IL-10	1:300	Anti-goat	SC-365858 Mouse Polyclonal; Sta Cruz Biotechnology, CA, USA
IL-13	1:200	Anti-mouse	SC-393365 Goat Polyclonal; Sta Cruz Biotechnology, CA, USA
MMP-9	1:300	Anti-mouse	SC-393859 Rabbit Polyclonal;Sta Cruz Biotechnology, CA, USA
MMP-12	1:300	Anti-rabbit	SC-30072 Rabbit Polyclonal; Sta Cruz Biotechnology, CA, USA
TIMP-1	1:400	Anti-rabbit	MS- MAB Mouse Polyclonal; LifeSpan BioScien, Inc, WA, USA
TGF-β	1:500	Anti-mouse	SC-146 Rabbit Polyclonal; Sta Cruz Biotechnology, CA, USA
iNOS	1:400	Anti-rabbit	RB-9242 Mouse Polyclonal; Neomarkers, Fremont, CA, USA
NF-kB	1:400	Anti-mouse	SC-8008 Mouse Polyclonal; Sta Cruz Biotechnology, CA, USA

### Morphometric analysis

#### Positive cell counting in the airway and alveolar septum

Morphometric analysis was conducted using a light microscope (E200MV, Nikon Corporation, Tokyo, Japan) equipped with an eyepiece graticule consisting of 50 lines and 100 points, corresponding to a total area of 10^4^µm². Cell quantification was performed using the point-counting technique with a graticule containing straight lines, as previously described. For airway analysis, the reticle was positioned along the bronchoalveolar axis, beginning at the epithelial base. Three airways were randomly selected per animal, and three fields were analyzed within each airway. The number of points overlapping positively stained cells in the airway wall was divided by the total number of points assessed. For alveolar septal analysis, the reticle was positioned over this region, and ten randomly selected fields were evaluated. The number of points intersecting positively stained cells on the alveolar septa was divided by the total number of points intersecting the alveolar structure. All assessments were conducted at 1.000× magnification (18).

### Cryopreservation of tissues and RNA extraction

For molecular studies, the right lungs of the animals were collected, washed with 0.9% saline solution, and immersed in liquid nitrogen in cryopreservation tubes. The samples were then stored in a -70 °C freezer until further analysis. Total RNA from the samples was isolated using an acidic commercial solution (Trizol™ Reagent – Invitrogen Life Technologies). Approximately 30 mg of lung tissue from the right lung of each animal was separated and weighed on an analytical balance. This tissue, still frozen in liquid nitrogen, was subjected to impact pulverization, resulting in a fine powder, which was then transferred to a plastic microtube.

For total RNA extraction, the RNeasy Kit (Qiagen, USA) was used in combination with Trizol™ (ThermoFisher, USA). Each sample was lysed with 1 mL of Trizol™, followed by vortexing for 20 seconds. After lysis, 200 μL of chloroform per sample was added for phase separation, mixed vigorously for 15 seconds, and incubated at room temperature for 2–3 minutes. Samples were then centrifuged at 12,000 × g for 15 minutes at 4 °C to promote separation of the aqueous and organic phases. The aqueous phase, containing RNA, was carefully transferred to a new silica column from the RNeasy kit. The lysate was applied to the column, where RNA selectively bound in the presence of a high salt concentration, resulting in a recovered volume of approximately 500 μL in the aqueous extraction phase. Washing steps were performed using the RWP and RPE buffers provided with the kit to remove contaminants such as proteins and DNA. The purified RNA was eluted from the column with 50 μL of nuclease-free water and stored in a -80 °C freezer.

### Reverse transcription

The concentration and purity of total RNA were evaluated using spectrophotometry with the NanoDrop 2000 spectrophotometer.

Reverse transcription was performed using the High-Capacity cDNA Reverse Transcription Kit (Applied Biosystems, USA) with 500 ng of total RNA. The reverse transcription reaction mixture included reverse transcription buffer, dNTPs, random primers, MultiScribe™ reverse transcriptase, and RNase inhibitor, following the manufacturer’s recommended proportions. The reaction mixture was incubated at 25 °C for 10 minutes, followed by 37 °C for 120 minutes for cDNA synthesis. The reverse transcriptase was then inactivated by heating at 85 °C for 5 minutes. The resulting cDNA was subsequently used for gene expression analyses.

### Real-time PCR

The real-time PCR reaction was performed using TaqMan Gene Expression components (ThermoFisher, USA) for mice (*Mus musculus*), according to the manufacturer’s instructions. TaqMan probes (ThermoFisher, USA) were obtained for the analysis of the following genes: *IL-4* (Mm00445259_m1), *IL-5* (Mm00439646_m1), *IL-6* (Mm00446190_m1), *IL-13* (Mm00434204_m1), and the housekeeping gene *ACTB* (Mm04394036_g1).

### Data analysis

Data were analyzed using SigmaPlot 11.0 software (Systat Software, SPSS Inc., USA). Results are expressed as means ± standard error (SE), and the graphs are presented as bar charts. Group differences were assessed by one-way ANOVA, followed by the Holm–Sidak *post hoc* test for multiple comparisons when significant main effects were observed. A *p*-value < 0.05 was considered statistically significant.

## Results

### Effect of melatonin on airway hyperresponsiveness

The respiratory mechanics in the animals after challenge with methacholine in the five experimental groups are presented in **Figure 1**. Compared to the control SAL group, the OVA group showed increased %Rrs, %Ers, %Gtis, and %Htis across all comparisons. Melatonin significantly attenuated airway hyperresponsiveness in the OVA model, reducing Rrs (*p* < 0.001), Ers (*p* < 0.01), Gtis (*p* < 0.001), and Htis (*p* < 0.05). In contrast, melatonin had no significant effect on airway responsiveness in the elastase-induced model.

**Figure 1:**
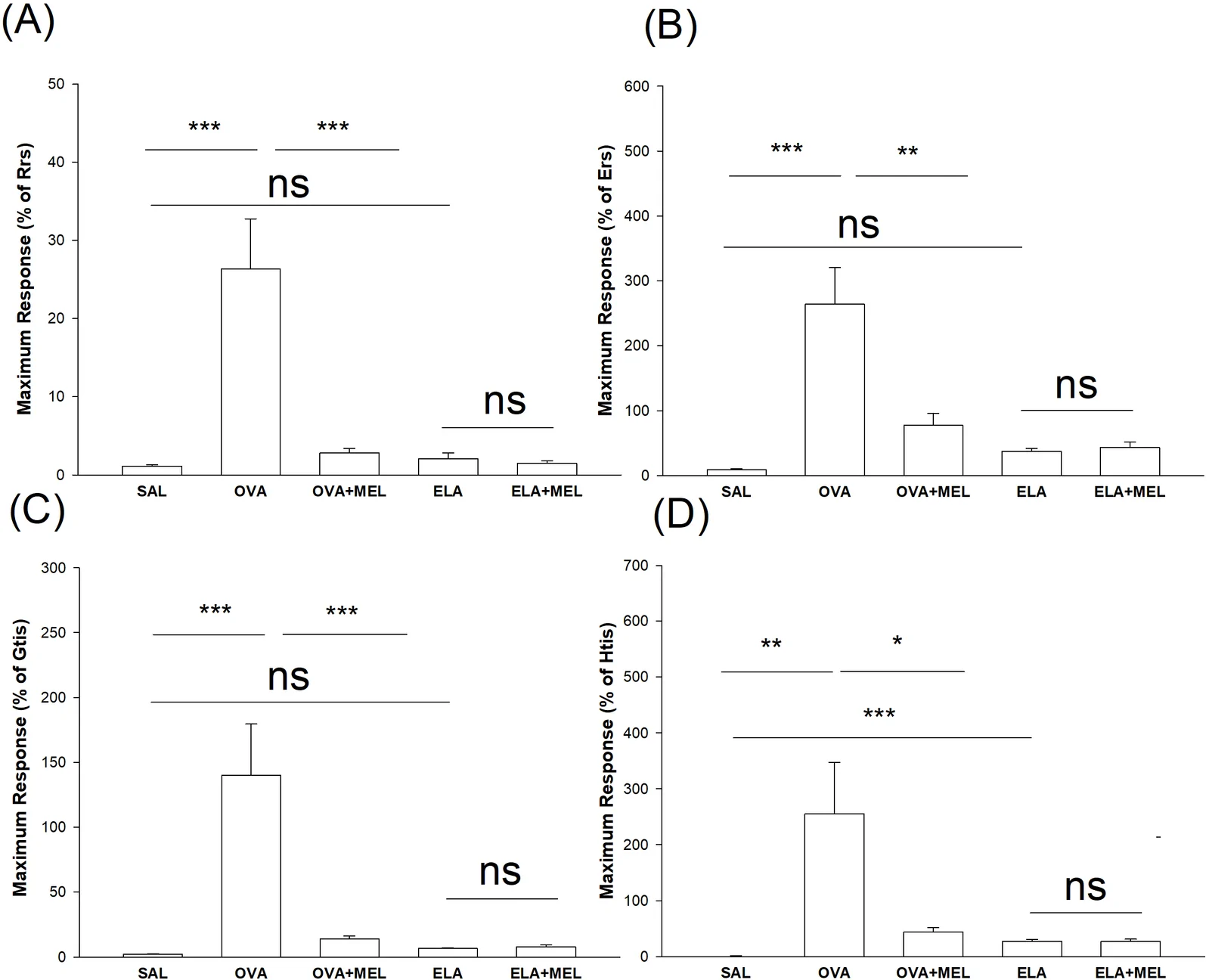
Airway hyperresponsiveness. Results of airway hyperresponsiveness in relation to respiratory mechanics parameters (Rrs, Ers, Gtis, and Htis). The mean ± standard error of the maximum response after methacholine challenge for all experimental groups. Values are presented as percentages. **(A)** Resistance (Rrs); **(B)** Elastance (Ers); **(C)** Resistance of smaller airways or tissues (Gtis) and **(D)** elastance of lung tissues (Htis). ns,not significant (p>0.05), *p<0.05, **p<0.01, ***p<0.001.

### Effect of melatonin on bronchoalveolar lavage fluid

The total cell count and the differential count of eosinophils, neutrophils, lymphocytes, and macrophages in the bronchoalveolar lavage fluid are shown in **Figure 2** and expressed as 10^4^ cells/mL.

**Figure 2:**
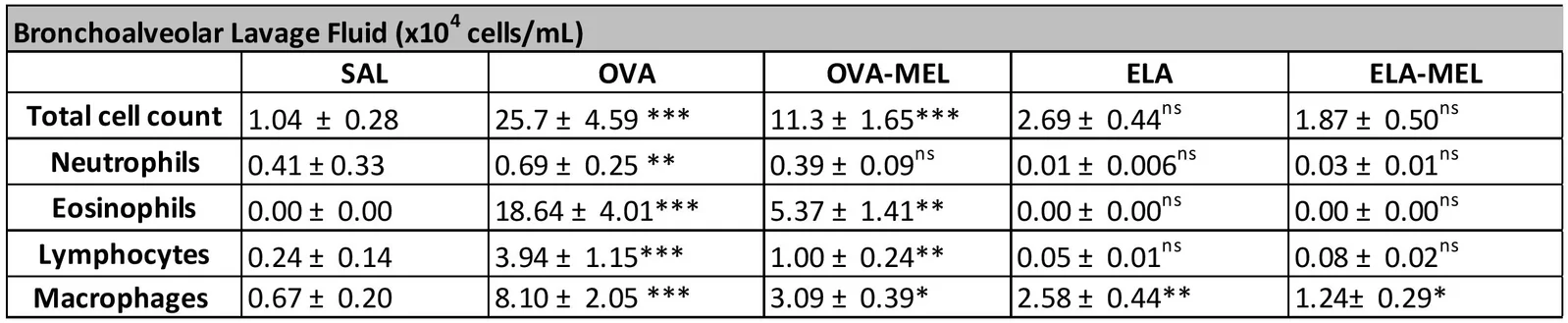
Absolute values of the number of cells in bronchoalveolar lavage fluid (BALF). The total cells, neutrophils, eosinophils, lymphocytes and macrophages are expressed as 10^4^cells/mL. *p<0.05, **p<0.01, ***p<0.001.

Melatonin significantly reduced inflammatory cell counts in the OVA model. Total cell counts were markedly reduced in the OVA+MEL group compared with the OVA group (*p* < 0.001), although they remained higher than in the SAL group (*p* < 0.01). Macrophage (*p* < 0.05), eosinophil (*p* < 0.01), and lymphocyte (*p* < 0.01) counts were also significantly reduced following melatonin treatment compared with the OVA group. Neutrophil counts were significantly increased in the OVA group compared with SAL (*p* < 0.01), but melatonin did not significantly modify this parameter. In the elastase-induced model, melatonin significantly reduced macrophage counts (*p* < 0.05), whereas no significant differences were observed in total cells, neutrophils, or lymphocytes. Eosinophils were absent in all elastase-treated groups.

### Effect of the melatonin on inflammation

Cytokine expression was significantly altered across the experimental groups. In the alveolar septum, IL-2 expression was higher in OVA and OVA+MEL than in SAL (both *p* < 0.001), with further elevation in OVA+MEL compared with OVA (*p* < 0.01). In ELA, IL-2 expression was higher in ELA+MEL than in both SAL and ELA (both *p* < 0.001), whereas ELA did not differ from SAL (*p* > 0.05). In the airways, IL-2 expression was higher in OVA and OVA+MEL than in SAL (*p* < 0.001 and *p* < 0.01, respectively), with no difference between these groups (*p* > 0.05). In ELA, IL-2 expression was higher in ELA than in SAL (*p* < 0.01), with no other significant differences (*p* > 0.05).

IL-4 expression was higher in OVA than in SAL and OVA+MEL in the alveolar septum (*p* < 0.001 and *p* < 0.05, respectively), whereas OVA+MEL did not differ from SAL (*p* > 0.05). In ELA, IL-4 expression was higher in ELA+MEL than in SAL and ELA (*p* < 0.01 and *p* < 0.05, respectively), with no difference between ELA and SAL (*p* > 0.05). In the airways, IL-4 expression was higher in both OVA and OVA+MEL than in SAL (both *p* < 0.001), and higher in OVA than in OVA+MEL (*p* < 0.01). In ELA, IL-4 expression was higher in both ELA and ELA+MEL than in SAL (*p* < 0.001 and *p* < 0.01, respectively), with no difference between the two ELA groups (*p* > 0.05).

IL-5 expression was higher in OVA than in SAL and OVA+MEL in both compartments (both *p* < 0.001), whereas OVA+MEL did not differ from SAL (*p* > 0.05). In ELA, IL-5 expression was higher in ELA than in SAL and was reduced following melatonin treatment in the alveolar septum (*p* < 0.001), with no difference between ELA+MEL and SAL (*p* > 0.05). In the airways, IL-5 expression differed among all groups (all *p* < 0.001), with melatonin reducing expression compared with ELA but not restoring levels to those observed in SAL.

IL-6 expression was higher in OVA and ELA than in SAL and the respective melatonin-treated groups in both compartments (all *p* < 0.001), whereas the melatonin-treated groups did not differ from SAL (*p* > 0.05).

For IL-10, OVA showed higher expression than SAL and OVA+MEL in the alveolar septum (both *p* < 0.001), with no difference between OVA+MEL and SAL (*p* > 0.05). In ELA, IL-10 expression was higher in ELA than in SAL and ELA+MEL (both *p* < 0.001), and ELA+MEL was higher than SAL (*p* < 0.05). In the airways, IL-10 expression was higher in OVA than in SAL and OVA+MEL (both *p* < 0.001), with OVA+MEL also higher than SAL (*p* < 0.001). In ELA, IL-10 expression was higher in ELA than in SAL (*p* < 0.001) and ELA+MEL (*p* < 0.05), whereas ELA+MEL did not differ from SAL (*p* > 0.05).

IL-13 expression was higher in OVA than in SAL in the alveolar septum (*p* < 0.001) and was reduced by melatonin (*p* < 0.01), although remaining higher than SAL (*p* < 0.01). In ELA, IL-13 expression was higher in ELA than in SAL and ELA+MEL (*p* < 0.001 and *p* < 0.01, respectively), with no difference between ELA+MEL and SAL (*p* > 0.05). In the airways, IL-13 expression was higher in OVA than in SAL and OVA+MEL (both *p* < 0.001), with OVA+MEL remaining higher than SAL (*p* < 0.01). In ELA, IL-13 expression was higher in ELA than in SAL and ELA+MEL (both *p* < 0.001), with no difference between ELA+MEL and SAL (*p* > 0.05).

Qualitative and quantitative analyses in IL-5 and IL-13 are shown in **Figures 3** and **4**.

**Figure 3:**
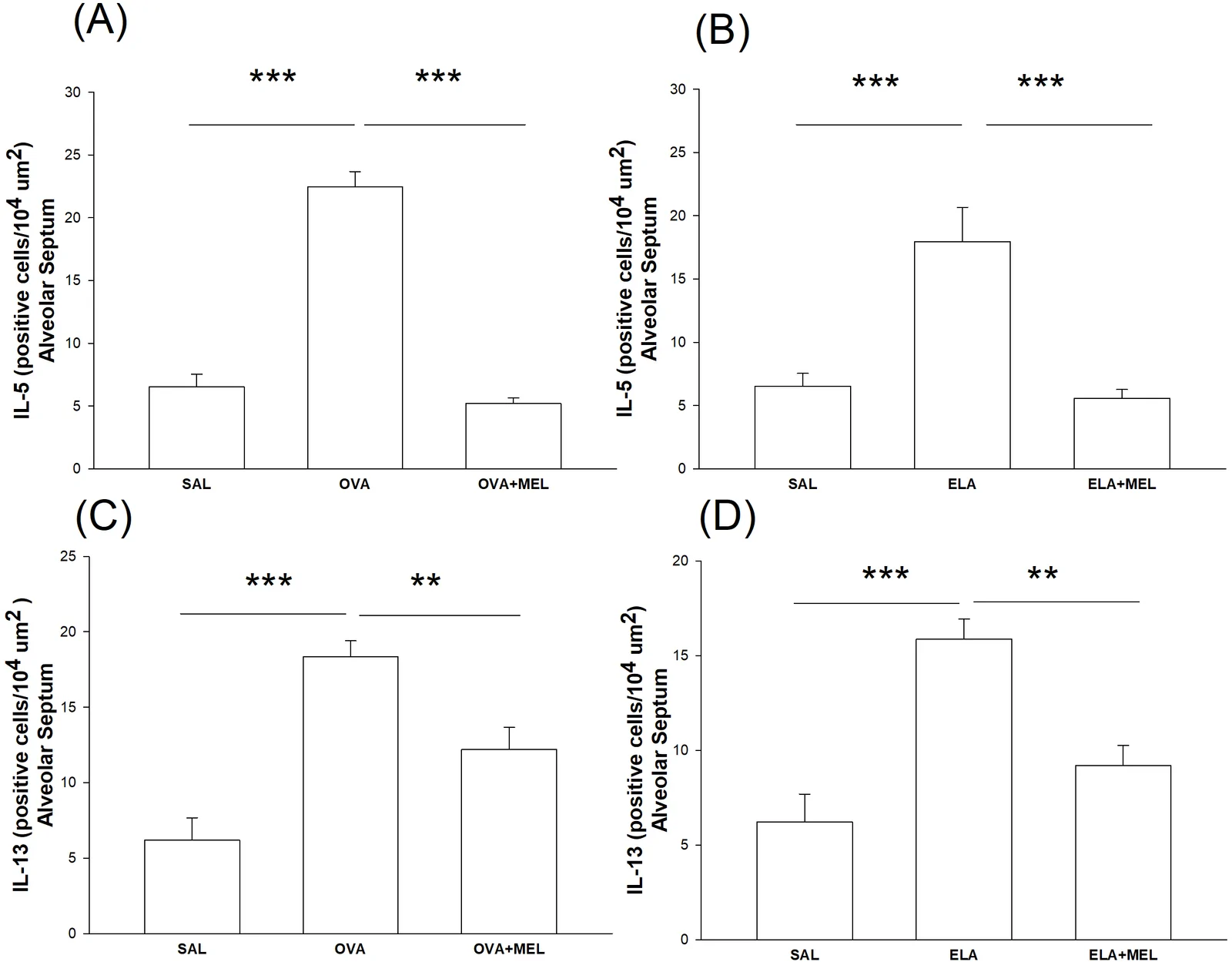
Inflammation markers in alveolar septum. The graphs illustrate the expression of the inflammatory markers IL-5 and IL-13 in the alveolar septum. **(A)** Graphical Illustration of IL-5 in the Alveolar Septa of the SAL, OVA, and OVA+MEL Groups **(B)** Graphical Illustration of IL-5 in the Alveolar Septa of the SAL, ELA, and ELA+MEL Groups **(C)** Graphical Illustration of IL-13 in the Alveolar Septa of the SAL, OVA, and OVA+MEL Groups **(D)** Graphical Illustration of IL-13 in the Alveolar Septa of the SAL, ELA, and ELA+MEL Groups. ns, not significant (p > 0.05), *p< 0.05, **p < 0.01, ***p <0.001.

**Figure 4:**
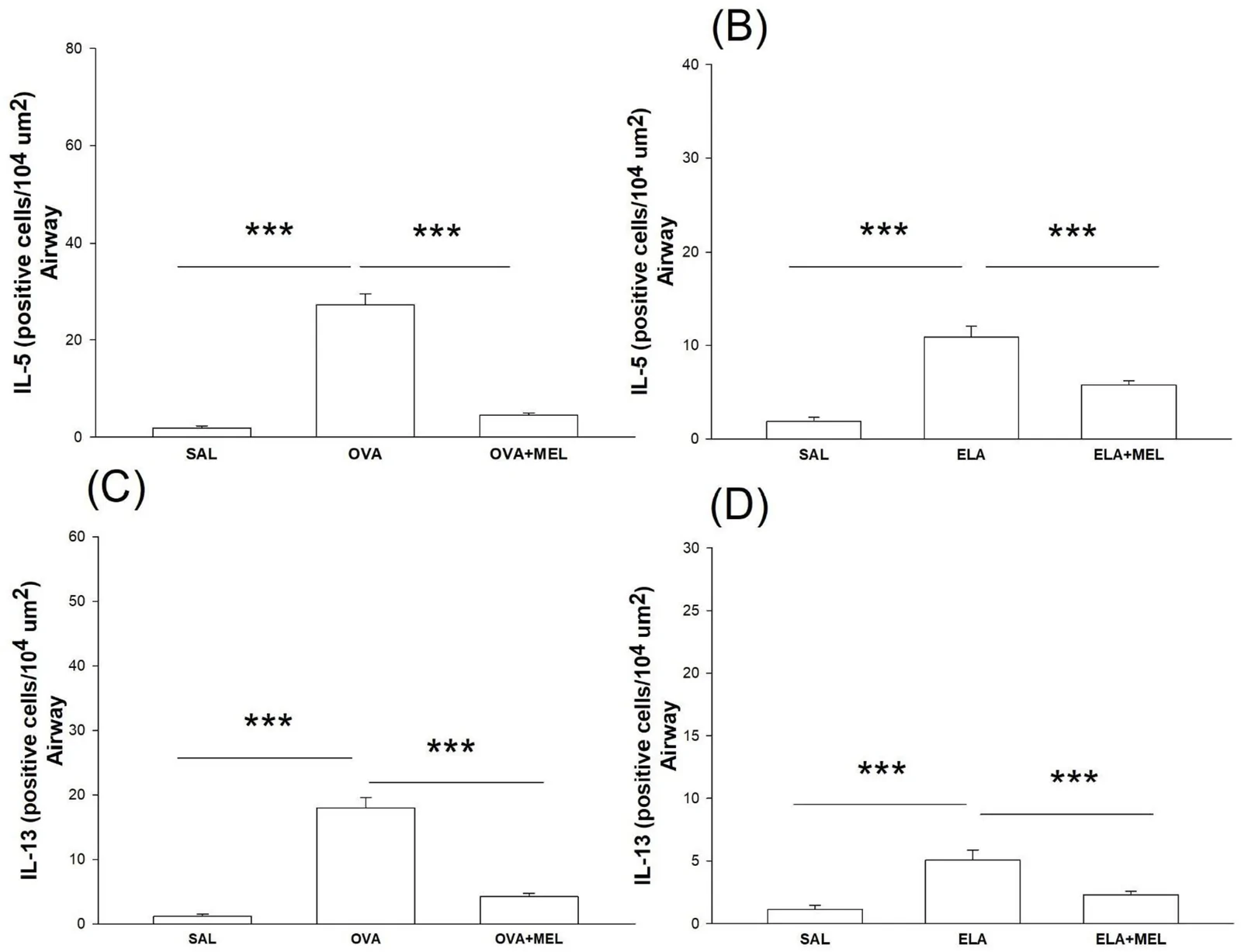
Airway inflammation markers. The graphs illustrate the expression of the inflammatory markers IL-5 and IL-13 in the airway. **(A)** Graphical Illustration of IL-5 in the airway of the SAL, OVA, and OVA+MEL Groups **(B)** Graphical Illustration of IL-5 in the airway of the SAL, ELA, and ELA+MEL Groups **(C)** Graphical Illustration of IL-13 in the airway of the SAL, OVA, and OVA+MEL Groups **(D)** Graphical Illustration of IL-13 in the airway of the SAL, ELA, and ELA+MEL Groups. ns, not significant (p > 0.05), *p< 0.05, **p < 0.01, ***p <0.001.

### Effect of melatonin on extracellular matrix remodeling

MMP-9 expression was significantly altered in both experimental models. In the alveolar septum, MMP-9 expression was higher in OVA than in both SAL and OVA+MEL (*p* = 0.001 and *p* < 0.001, respectively), whereas OVA+MEL did not differ from SAL (*p* > 0.05). In ELA, MMP-9 expression was significantly reduced following melatonin treatment compared with ELA (*p* < 0.001), while no significant differences were observed between ELA and SAL or between ELA+MEL and SAL (*p* > 0.05). In the airways, MMP-9 expression was higher in OVA than in both SAL and OVA+MEL (both *p* < 0.001), with no difference between OVA+MEL and SAL (*p* > 0.05). Similarly, in ELA, MMP-9 expression was higher in ELA than in both SAL and ELA+MEL (both *p* < 0.001), whereas ELA+MEL did not differ from SAL (*p* > 0.05). Photomicrographs of the results of the immunohistochemical analysis of the extracelluar matrix remodeling in the alveolar septa to dectect MMP-9 (**Figure 5**).

**Figure 5:**
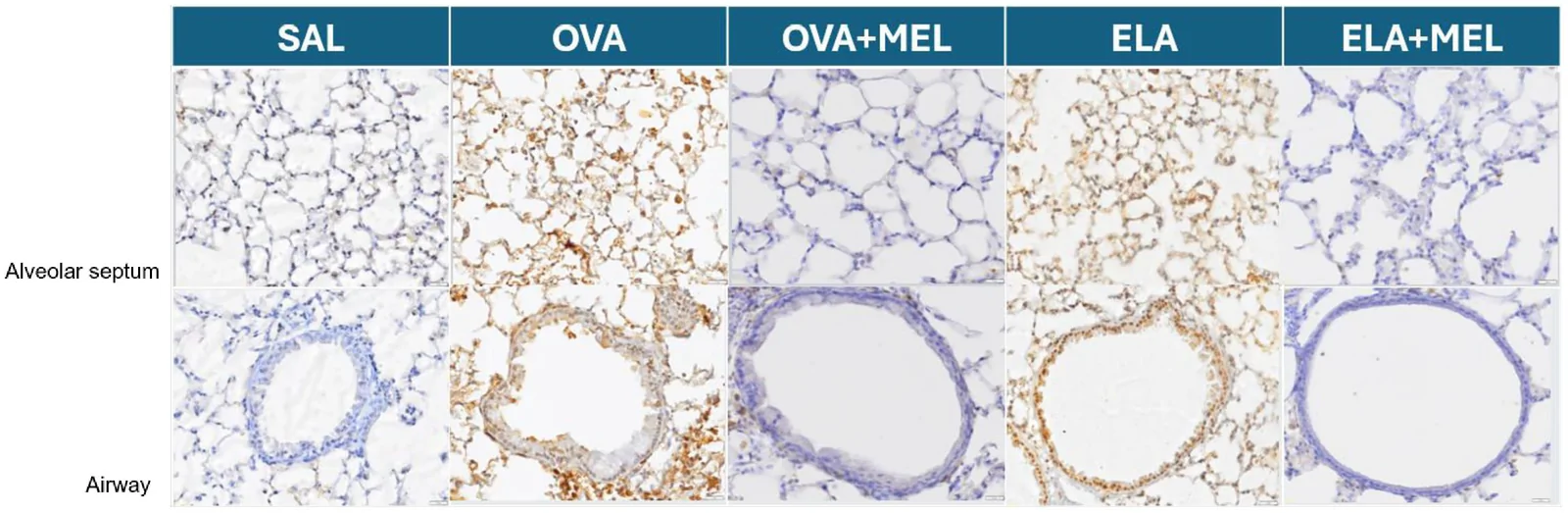
Representative photomicrographs of MMP-9 immunohistochemical expression in the alveolar septum and airway, reflecting extracellular matrix remodeling. Images were acquired at 400× magnification, with a scale bar of 20 μm. Images from the SAL, OVA, OVA+MEL, ELA, and ELA+MEL experimental groups.

MMP-12 expression was also significantly altered. In the alveolar septum, MMP-12 expression was higher in OVA than in both SAL and OVA+MEL (both *p* < 0.001), with no difference between OVA+MEL and SAL (*p* > 0.05). In ELA, MMP-12 expression was higher in ELA than in SAL (*p* < 0.01) and ELA+MEL (*p* < 0.05), whereas ELA+MEL did not differ from SAL (*p* > 0.05). In the airways, MMP-12 expression was higher in OVA than in both SAL and OVA+MEL (both *p* < 0.001), with no difference between OVA+MEL and SAL (*p* > 0.05). In ELA, MMP-12 expression was also higher in ELA than in both SAL and ELA+MEL (both *p* < 0.001), whereas ELA+MEL did not differ from SAL (*p* > 0.05).

TIMP-1 expression was significantly reduced following melatonin treatment in both models and compartments. In the alveolar septum, TIMP-1 expression was lower in OVA+MEL than in OVA (*p* < 0.01), although levels remained higher than SAL (*p* < 0.05). In ELA, TIMP-1 expression was higher in ELA than in both SAL (*p* < 0.01) and ELA+MEL (*p* < 0.01), whereas ELA+MEL did not differ from SAL (*p* > 0.05). In the airways, TIMP-1 expression was reduced in OVA+MEL compared with OVA (*p* < 0.001), although levels remained higher than SAL (*p* < 0.05). Similarly, in ELA, TIMP-1 expression was reduced following melatonin treatment compared with ELA (*p* < 0.01), but remained higher than SAL (*p* < 0.05).

TGF-β expression was significantly altered in both models. In the alveolar septum, TGF-β expression was higher in OVA than in SAL (*p* < 0.01), whereas the reduction observed in OVA+MEL compared with OVA was not significant (*p* > 0.05); however, TGF-β expression remained higher in OVA+MEL than in SAL (*p* < 0.05). In ELA, TGF-β expression was higher in ELA+MEL than in both ELA and SAL (both *p* < 0.001), whereas ELA did not differ from SAL (*p* > 0.05). In the airways, TGF-β expression was reduced in OVA+MEL compared with OVA (*p* < 0.001), although levels remained higher than SAL (*p* < 0.01). In ELA, TGF-β expression was higher in both ELA and ELA+MEL than in SAL (both *p* < 0.001), with no significant difference between ELA+MEL and ELA (*p* > 0.05).

### Effect of melatonin on oxidative stress and signaling pathways

Quantification of iNOS- and NF-κB-positive cell expression in the airways and alveolar septa is presented in **Figure 6**. iNOS expression was significantly increased in both disease models and was reduced following melatonin treatment. In the alveolar septum, iNOS expression was higher in OVA and ELA than in their respective SAL groups (both *p* < 0.001) and was significantly reduced by melatonin in both models (both *p* < 0.001), with no difference between the melatonin-treated and SAL groups (*p* > 0.05). In the airways, iNOS expression was similarly higher in OVA and ELA than in SAL (both *p* < 0.001) and was reduced following melatonin treatment in both models (both *p* < 0.001). In OVA, iNOS expression in OVA+MEL did not differ from SAL (*p* > 0.05), whereas in ELA, expression remained significantly different from SAL (*p* < 0.05).

**Figure 6:**
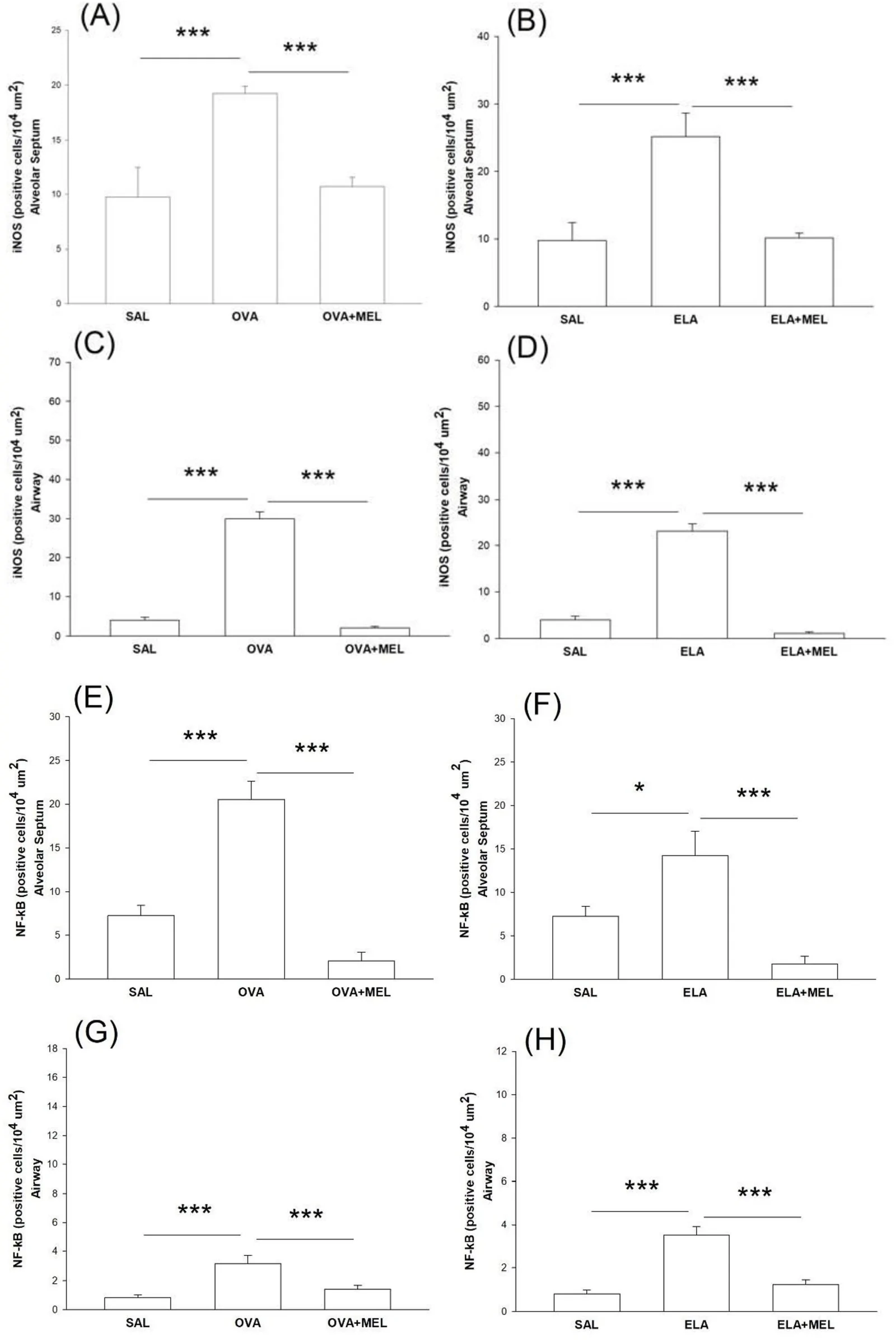
Oxidative stress markers. The graphs illustrate iNOS and NF-κB expression in the SAL, OVA, OVA+MEL, ELA, and ELA+MEL groups. **(A)** iNOS expression in the alveolar septum of the SAL, OVA, and OVA+MEL groups. **(B)** iNOS expression in the alveolar septum of the SAL, ELA, and ELA+MEL groups. **(C)** iNOS expression in the airway of the SAL, OVA, and OVA+MEL groups. **(D)** iNOS expression in the airway of the SAL, ELA, and ELA+MEL groups. **(E)** NF-κB expression in the alveolar septum of the SAL, OVA, and OVA+MEL groups. **(F)** NF-κB expression in the alveolar septum of the SAL, ELA, and ELA+MEL groups. **(G)** NF-κB expression in the airway of the SAL, OVA, and OVA+MEL groups. **(H)** NF-κB expression in the airway of the SAL, ELA, and ELA+MEL groups. ns, not significant (p > 0.05), *p< 0.05, **p < 0.01, ***p <0.001.

NF-κB expression was also significantly altered by the experimental conditions. In the alveolar septum, NF-κB expression was higher in OVA than in SAL (*p* < 0.001) and was reduced following melatonin treatment (*p* < 0.001), reaching levels significantly lower than SAL (*p* < 0.05). In ELA, NF-κB expression was higher than SAL (*p* < 0.05) and was reduced by melatonin (*p* < 0.001), with no significant difference between ELA+MEL and SAL (*p* > 0.05). In the airways, NF-κB expression was higher in both OVA and ELA than in SAL (both *p* < 0.001) and was reduced following melatonin treatment in both models (both *p* < 0.001), with no significant differences between the melatonin-treated and SAL groups (*p* > 0.05).

## Discussion

The present study demonstrated that melatonin exerted significant therapeutic effects in experimental models of asthma and COPD by improving airway hyperresponsiveness, reducing airway inflammation, and attenuating markers associated with pulmonary remodeling. These effects were accompanied by decreased expression of key pro-inflammatory mediators, including Th2 cytokines, NF-κB, inducible nitric oxide synthase (iNOS), matrix metalloproteinases, and factors involved in extracellular matrix remodeling, suggesting that the beneficial effects of melatonin result from the integrated modulation of multiple cellular pathways involved in the pathophysiology of these diseases.

Among the main findings of the present study was the improvement in mechanical airway hyperresponsiveness observed in the experimental asthma model. Melatonin treatment significantly reduced the maximal methacholine response, as evidenced by decreases in respiratory system resistance (Rrs), respiratory system elastance (Ers), tissue damping (Gtis), and tissue elastance (Htis), indicating improvements in both proximal airway resistance and the mechanical properties of the distal airways and alveolar walls. These findings suggest that melatonin effectively attenuated airway hyperresponsiveness, one of the major pathophysiological hallmarks of asthma, which is directly associated with variable airflow limitation and disease severity (1).

Airway hyperresponsiveness is a major determinant of asthma symptoms and is strongly associated with the extent of airway inflammation and bronchial remodeling. Clinical studies have demonstrated that patients with more severe asthma exhibit greater bronchoconstrictor responses to bronchial challenge agents, reflecting persistent structural and inflammatory changes within the airways (1). In this context, the functional improvement observed in the present study is unlikely to represent an isolated effect of melatonin on airway smooth muscle. Rather, it most likely reflects the simultaneous modulation of multiple mechanisms involved in the pathophysiology of asthma, including the attenuation of inflammation, oxidative stress, and pulmonary remodeling.

Conversely, as expected, melatonin treatment did not produce significant changes in the methacholine response in the elastase-induced lung injury model. This finding is consistent with the pathophysiological characteristics of this experimental model, in which pulmonary parenchymal destruction and loss of elastic recoil are the primary determinants of airflow limitation, in contrast to asthma, where airway hyperresponsiveness represents the predominant functional abnormality. These findings are also consistent with clinical studies involving patients with COPD, in which melatonin administration improved dyspnea and quality of life without producing significant changes in spirometric parameters (15). Collectively, these results suggest that the beneficial effects of melatonin in COPD are more likely attributable to the modulation of inflammation and oxidative stress than to direct effects on pulmonary mechanics.

Supporting this interpretation, experimental studies have demonstrated that melatonin reduces the expression of connective tissue growth factor (CTGF), decreases mucus production, and attenuates airway remodeling in experimental models of asthma (10). Considering that pulmonary remodeling directly contributes to airway hyperresponsiveness, it is likely that the functional improvement observed in the present study results from the integrated effects of melatonin on both the inflammatory and structural components of the disease. This hypothesis is further supported by the findings obtained for the inflammatory and remodeling markers evaluated in the present study.

The improvement in pulmonary mechanics was accompanied by a marked reduction in airway inflammation, as evidenced by decreased inflammatory cell infiltration in bronchoalveolar lavage fluid (BALF) and by the modulation of key cytokines involved in the adaptive immune response. In experimental models of asthma, melatonin treatment significantly reduced the total number of cells, eosinophils, lymphocytes, and macrophages in bronchoalveolar lavage fluid, indicating reduced recruitment of inflammatory cells into the lung tissue. These findings are consistent with previous experimental studies demonstrating that melatonin reduces inflammatory cell infiltration and mucus production, thereby attenuating pulmonary inflammation in experimental models of allergic asthma (8, 11).

The reduction in eosinophilic infiltration observed in the present study is of particular relevance, as eosinophilic inflammation is one of the hallmark features of allergic asthma and is closely associated with airway hyperresponsiveness, epithelial damage, and airway remodeling. This finding was accompanied by a consistent reduction in the expression of cytokines characteristic of the Th2 immune response, suggesting that melatonin modulates key mechanisms underlying allergic airway inflammation.

The type 2 immune response plays a pivotal role in the pathophysiology of asthma by promoting Th2 lymphocyte differentiation, IgE production, eosinophil recruitment and activation, mucus hypersecretion, and airway hyperresponsiveness. Among the major cytokines involved in this process are IL-4, IL-5, and IL-13, whose coordinated actions perpetuate allergic inflammation and contribute to the development of pulmonary remodeling (21–25). In the present study, melatonin treatment significantly reduced the expression of all three cytokines, as demonstrated by both immunohistochemical and quantitative real-time PCR (qRT-PCR) analyses, indicating consistent suppression of the Th2 immune response.

IL-4 is one of the principal drivers of naïve T-cell polarization toward the Th2 phenotype and directly participates in IgE production, adhesion molecule expression, eotaxin production, and goblet cell hyperplasia (21, 22, 24). The reduction in IL-4 expression observed following melatonin treatment suggests attenuation of the early activation of the Th2 immune response, a finding consistent with previous studies reporting similar effects in experimental models of asthma treated with melatonin (26).

Similarly, the reduction in IL-5 expression is of considerable biological significance, as this cytokine is essential for eosinophil differentiation, maturation, recruitment, and survival (26, 27). Given that eosinophils are major effector cells in allergic inflammation, the decrease in IL-5 expression observed in the present study provides a mechanistic explanation for the concomitant reduction in eosinophil numbers detected in bronchoalveolar lavage fluid. This interpretation is consistent with previous experimental studies demonstrating that reduced IL-5 expression limits eosinophil recruitment, decreases tissue damage, and attenuates airway remodeling (28, 29).

IL-13 is another key mediator of the Th2 immune response and plays an important role in mucus production, airway hyperresponsiveness, and pulmonary remodeling (30). The reduction in its expression, observed by both immunohistochemistry and quantitative real-time PCR (qRT-PCR), further supports the ability of melatonin to simultaneously modulate multiple components of the allergic response. Considering that IL-4, IL-5, and IL-13 act in a coordinated manner, the concomitant reduction in these cytokines suggests an overall suppression of the type 2 inflammatory response rather than an effect restricted to a single inflammatory mediator.

In addition to modulating Th2 cytokines, melatonin also reduced IL-6 expression, a pleiotropic cytokine produced primarily by macrophages, fibroblasts, and endothelial cells in response to inflammatory stimuli (31). Although IL-6 exerts complex immunoregulatory functions, elevated IL-6 expression has been consistently associated with the maintenance of chronic inflammation in respiratory diseases. The reduction observed by both immunohistochemistry and qRT-PCR further reinforces the anti-inflammatory effects of melatonin and demonstrates that its actions are not exclusively limited to the modulation of the Th2 immune response.

The interpretation of the reduction in IL-10 expression deserves particular consideration. Although this cytokine is widely recognized for its anti-inflammatory properties, accumulating evidence indicates that its expression is highly dependent on the immunological context (32–36). In experimental models of allergic inflammation, increased IL-10 expression frequently occurs as a compensatory mechanism in response to intense inflammatory activation, without necessarily reflecting effective resolution of the immune response (37). Therefore, the reduction in IL-10 expression observed following melatonin treatment most likely represents a consequence of the overall attenuation of inflammation rather than a loss of regulatory mechanisms. This interpretation is supported by the simultaneous reduction in eosinophilic infiltration, Th2 cytokine expression, and the other inflammatory markers evaluated in the present study.

Collectively, these findings demonstrate that melatonin promotes coordinated modulation of the pulmonary inflammatory response by simultaneously reducing inflammatory cell recruitment and the expression of key cytokines involved in maintaining the type 2 immune response. This integrated effect provides a consistent mechanistic explanation for the functional improvement observed in the treated animals and establishes the basis for the effects of melatonin on molecular pathways associated with oxidative stress and pulmonary remodeling, which are discussed in the following sections.

The anti-inflammatory effects observed in the present study are likely attributable to the ability of melatonin to modulate key mechanisms involved in the interplay between oxidative stress and the inflammatory response. These processes are currently recognized as closely interconnected components of the pathophysiology of asthma and COPD, establishing a positive feedback loop in which excessive production of reactive species promotes the activation of inflammatory pathways, while inflammation further enhances oxidant production. Within this context, melatonin is distinguished by its pleiotropic actions, which extend beyond the direct scavenging of reactive oxygen species (ROS) and reactive nitrogen species (RNS), also acting as an important modulator of intracellular signaling pathways involved in inflammation (38–43).

Among these pathways, nuclear factor-kappa B (NF-κB) plays a central role in the regulation of the inflammatory response. NF-κB activation is highly sensitive to the cellular redox state and induces the transcription of numerous genes involved in inflammation, including pro-inflammatory cytokines, adhesion molecules, chemokines, matrix metalloproteinases, and inducible nitric oxide synthase (iNOS) (41, 44, 45). Under physiological conditions, NF-κB remains inactive in the cytoplasm through its association with the inhibitory protein IκBα. However, under conditions of oxidative stress, degradation of this inhibitory protein occurs, leading to the translocation of NF-κB into the nucleus and the initiation of an inflammatory cascade that contributes to the perpetuation of pulmonary injury (41, 44, 46–49).

In the present study, the reduction in NF-κB expression observed following melatonin treatment, in both the experimental asthma model and the elastase-induced lung injury model, suggests that melatonin interferes with one of the principal mechanisms responsible for sustaining chronic inflammation. These findings are consistent with experimental evidence demonstrating that melatonin stabilizes the IκBα protein, thereby preventing NF-κB activation and nuclear translocation and consequently reducing the expression of several pro-inflammatory mediators (44, 46–49). Thus, in addition to directly reducing the availability of reactive species, melatonin limits the activation of one of the major pathways responsible for amplifying the inflammatory response.

This modulation becomes particularly relevant when considered together with the findings related to iNOS. The expression of this enzyme is largely dependent on NF-κB activation and is associated with sustained nitric oxide production during inflammatory processes (50, 51). Although nitric oxide plays important physiological roles, its excessive production favors the formation of peroxynitrite (ONOO^−^) through its reaction with the superoxide anion, thereby enhancing nitrosative stress, lipid peroxidation, and cellular damage. Therefore, the concomitant reduction in NF-κB and iNOS expression observed in the present study suggests that melatonin simultaneously limits the activation of inflammatory pathways and the generation of reactive species capable of perpetuating pulmonary injury.

These findings reinforce that the antioxidant and anti-inflammatory properties of melatonin do not represent independent mechanisms but rather components of an integrated biological response. By reducing the availability of reactive species, melatonin decreases one of the major stimuli for NF-κB activation. Concurrently, inhibition of this pathway reduces the expression of iNOS and other inflammatory mediators, thereby limiting the formation of new reactive species and interrupting the amplification cycle between oxidative stress and inflammation. This interaction likely explains the simultaneous reduction in the different inflammatory markers observed in the present study.

Supporting this interpretation, recent preclinical evidence demonstrates that melatonin also modulates other mechanisms relevant to the pathophysiology of COPD. In addition to inhibiting the NF-κB signaling pathway, melatonin reduces the expression of transforming growth factor-beta 1 (TGF-β1), the NLRP3 inflammasome, oxidant production, and mucus hypersecretion, while simultaneously increasing sirtuin-1 (SIRT1) expression, enhancing endogenous antioxidant defenses, improving mitochondrial function, and attenuating endoplasmic reticulum dysfunction, all of which are recognized contributors to disease progression (47). Studies have also demonstrated that these effects depend, at least in part, on the activation of melatonin receptors MT1 and MT2 (48, 49). Furthermore, melatonin may modulate programmed cell death through the regulation of lipopolysaccharide-induced alterations in alanine, aspartate, and glutamate metabolic pathways, as well as through modulation of the PI3K/AKT signaling pathway and the activity of the α7 nicotinic acetylcholine receptor (52, 53). Collectively, these findings demonstrate that the biological effects of melatonin extend far beyond its direct antioxidant activity, involving broad regulation of redox homeostasis, cellular metabolism, and inflammatory signaling.

Considering the concomitant reduction in inflammatory cytokines, NF-κB, iNOS, and inflammatory cell infiltration observed in the present study, our findings suggest that melatonin promotes a global reprogramming of the pulmonary inflammatory response rather than merely modulating a single molecular target. This integrated action likely represents one of the principal mechanisms underlying the protective effects observed in the experimental models evaluated.

In addition to modulating the inflammatory response, our findings demonstrated that melatonin significantly affected markers associated with pulmonary remodeling, suggesting that its therapeutic effects extend to the preservation of the architecture of both the airways and the pulmonary parenchyma. Pulmonary remodeling is a fundamental feature of both asthma and COPD and results from the continuous interplay among chronic inflammation, oxidative stress, and alterations in extracellular matrix turnover. These changes include excessive collagen deposition, reorganization of elastic fibers, fibroblast activation, extracellular matrix degradation, and irreversible structural alterations of the lung, all of which contribute to the progressive decline in pulmonary function and the persistence of airway hyperresponsiveness (13, 54–58).

Among the major mediators involved in this process is matrix metalloproteinase-9 (MMP-9), an enzyme responsible for the degradation of several extracellular matrix components, including collagen, elastin, fibronectin, and gelatin (54, 55). In addition to its direct role in tissue remodeling, MMP-9 facilitates the migration of inflammatory cells into the lung tissue, thereby amplifying the inflammatory response and perpetuating structural damage. Experimental studies have demonstrated that MMP-9 expression is directly correlated with asthma severity (59).

In the present study, melatonin significantly reduced MMP-9 expression in both the experimental asthma model and the elastase-induced lung injury model. These findings are consistent with previous studies demonstrating that melatonin reduces both the expression and activity of MMP-9, thereby simultaneously attenuating airway inflammation and remodeling (11, 60, 61). In cigarette smoke-induced COPD models, melatonin has also been shown to significantly reduce neutrophilic infiltration, reactive oxygen species production, and the expression of IL-6, TNF-α, MUC5AC, and MMP-9, further supporting its role in preserving the structural integrity of lung tissue (62).

The biological significance of this finding becomes even more evident when considering the close relationship between MMP-9 and the Th2 inflammatory response. Previous studies have demonstrated that MMP-9 production is strongly influenced by the cytokines IL-4, IL-5, and IL-13 (63). Moreover, overexpression of this metalloproteinase exacerbates allergic inflammation and increases the levels of these cytokines, whereas its inhibition simultaneously reduces both the eosinophilic response and Th2 cytokine levels (60–64). This interaction is particularly relevant to the findings of the present study, as the reduction in MMP-9 expression occurred concomitantly with decreased IL-4, IL-5, and IL-13 expression, suggesting that melatonin disrupts positive feedback mechanisms between type 2 inflammation and pulmonary remodeling.

Another relevant finding was the reduction in MMP-12 expression following melatonin treatment. This matrix metalloproteinase, produced predominantly by macrophages, exhibits potent elastolytic activity and plays a central role in the development of emphysema by promoting the degradation of alveolar elastic fibers. The reduction in MMP-12 expression observed in the present study suggests that melatonin may also limit mechanisms involved in pulmonary parenchymal destruction, thereby extending its protective effects beyond the airways and contributing to the preservation of pulmonary structural integrity.

Extracellular matrix turnover depends not only on the activity of matrix metalloproteinases but also on the balance between these enzymes and their endogenous inhibitors, particularly tissue inhibitor of metalloproteinases-1 (TIMP-1). Although increased TIMP-1 expression initially represents a compensatory response to excessive proteolytic activity, its persistent expression promotes extracellular matrix accumulation and contributes to the development of fibrosis and airway wall thickening (65). In the present study, the reduction in TIMP-1 expression following melatonin treatment suggests a more effective restoration of extracellular matrix homeostasis, indicating that its effects are not limited to the inhibition of matrix metalloproteinases but also involve the normalization of the balance between proteolytic and antiproteolytic mechanisms.

These findings are further supported by the results obtained for transforming growth factor-beta (TGF-β), which is recognized as one of the principal mediators of pulmonary remodeling. This cytokine stimulates fibroblast proliferation, myofibroblast differentiation, collagen deposition, fibronectin synthesis, and proteoglycan production, thereby directly contributing to the development of fibrosis and airway wall thickening (66). The reduction in TGF-β expression observed following melatonin treatment, together with the decreased expression of MMP-9, MMP-12, and TIMP-1, suggests that melatonin acts at multiple stages of extracellular matrix remodeling. Consequently, its effects appear to involve both limiting excessive extracellular matrix degradation and preventing the excessive deposition of fibrotic components, thereby preserving pulmonary architecture.

When considered collectively, the findings related to MMP-9, MMP-12, TIMP-1, and TGF-β demonstrate that melatonin exerts a regulatory effect on the principal mechanisms involved in pulmonary remodeling. This modulation is likely integrated with the attenuation of inflammation and oxidative stress discussed above, as NF-κB, Th2 cytokines, and reactive species are important regulators of the expression of these molecules. Therefore, the effects of melatonin on pulmonary remodeling appear to represent a consequence of the restoration of pulmonary homeostasis rather than the isolated modulation of extracellular matrix proteins.

Collectively, the findings of the present study demonstrate that the therapeutic effects of melatonin cannot be attributed to the modulation of a single molecular target. Rather, our findings indicate that melatonin acts on an integrated network of mechanisms involved in the pathophysiology of asthma and COPD by simultaneously modulating the inflammatory response, oxidative stress, and pulmonary remodeling. The attenuation of the Th2 immune response, evidenced by the reduced expression of IL-4, IL-5, and IL-13, together with the decreased recruitment of eosinophils and other inflammatory cells into the bronchoalveolar lavage fluid, demonstrates a consistent attenuation of allergic inflammation. In parallel, the reduced expression of NF-κB and iNOS suggests that melatonin interferes with key inflammatory signaling pathways and redox homeostasis, thereby limiting the production of reactive species and interrupting the amplification cycle between oxidative stress and inflammation.

In addition to modulating the inflammatory response, the reduced expression of MMP-9, MMP-12, TIMP-1, and TGF-β demonstrates that melatonin also interferes with fundamental mechanisms involved in pulmonary remodeling. The concomitant regulation of these molecules suggests reduced dysregulated extracellular matrix degradation, decreased activation of fibrotic processes, and greater preservation of pulmonary architecture, thereby contributing to the functional improvement observed in the experimental asthma model. These findings reinforce the concept that inflammation, oxidative stress, and remodeling are biologically interconnected processes, in which modulation of one component directly influences the others.

This concept is further supported by evidence demonstrating that melatonin regulates multiple cellular pathways, including NF-κB, NLRP3, SIRT1, PI3K/AKT, and mechanisms associated with mitochondrial function and endoplasmic reticulum homeostasis (52, 53, 62, 67–70). Accordingly, its biological effects extend beyond its well-established antioxidant activity, characterizing melatonin as a molecule capable of restoring cellular homeostasis through the coordinated modulation of multiple signaling pathways involved in the maintenance of chronic inflammation.

Although limited to experimental models, the findings of the present study demonstrate the therapeutic potential of melatonin in inflammatory respiratory diseases. Its ability to simultaneously modulate functional, inflammatory, and structural alterations suggests that melatonin represents a promising therapeutic strategy for complex diseases such as asthma and COPD, in which multiple pathophysiological mechanisms operate in an integrated manner. Future studies are warranted to clarify the clinical relevance of these findings and to determine the optimal doses, routes of administration, and therapeutic protocols for its application in humans.

A major strength of the present study is its comprehensive experimental approach, which enabled an integrated evaluation of the effects of melatonin across multiple levels of the pathophysiology of chronic respiratory diseases. By combining pulmonary mechanics assessment, bronchoalveolar lavage fluid (BALF) analysis, immunohistochemistry, and quantitative real-time polymerase chain reaction (qRT-PCR), the present study demonstrated consistent effects of melatonin at the functional, cellular, histological, and molecular levels. Furthermore, the use of two distinct experimental models representing different pathophysiological mechanisms of asthma and COPD enhanced the translational relevance of the findings. Collectively, the results provide convergent evidence that melatonin coordinately modulates inflammatory, oxidative, and remodeling pathways, supporting the hypothesis that it acts as an important regulator of pulmonary homeostasis.

### Study limitations

This study has some limitations. Only a single dose of melatonin (15 mg/kg) administered intraperitoneally was evaluated, precluding dose–response analysis and limiting the direct clinical translation of the findings. In addition, the relatively short treatment period does not allow conclusions regarding the long-term effects of melatonin on airway and parenchymal remodeling. Finally, only male BALB/c mice were included, preventing the assessment of potential sex-related differences in therapeutic response. Future studies should investigate different doses, clinically relevant routes of administration, longer treatment protocols, and both sexes to further validate these findings.

## Conclusion

In summary, the experimental model employed consistently reproduced several classical pathological features of asthma and COPD, reinforcing the validity of the proposed design. The results obtained indicate that melatonin treatment demonstrated efficacy in modulating pulmonary hyperresponsiveness, as evidenced by the significant reduction in markers associated with inflammation, structural remodeling, and oxidative stress, playing a relevant role in the control of both asthma and COPD.

These findings corroborate previous literature reports highlighting the potential immunomodulatory and antioxidant effects of melatonin, suggesting that its use may represent a promising therapeutic strategy in the management of asthma and COPD. However, additional studies are necessary to elucidate the molecular mechanisms involved, determine the optimal dose–response relationship, and confirm the clinical applicability of these findings in human populations. Such future investigations may contribute to expanding the therapeutic arsenal available for chronic respiratory diseases, the control of which still represents a significant challenge for public health.

## Data Availability

The datasets presented in this study can be found in online repositories. The names of the repository/repositories and accession number(s) can be found in the article/**Supplementary Material**.
